# Assessment of HIF-1α and c-MYC in Breast Carcinoma and Their Association With Clinicopathological Parameters: A Cross-Sectional Study

**DOI:** 10.7759/cureus.100539

**Published:** 2025-12-31

**Authors:** Anibesh Mohapatra, Madhusmita Mohanty, Sabyasachi Parida, Urmila Senapati, Nihar Ranjan Mohanty

**Affiliations:** 1 Department of Pathology, Kalinga Institute of Medical Sciences, Bhubaneswar, IND; 2 Department of Surgical Oncology, Kalinga Institute of Medical Sciences (KIMS) Cancer Centre, Kalinga Institute of Medical Sciences, Bhubaneswar, IND; 3 Department of Radiology, Kalinga Institute of Medical Sciences, Bhubaneswar, IND

**Keywords:** breast carcinoma, clinicopathological parameters, c-myc proto-oncogene proteins, hif-1 alpha, immunohistochemistry

## Abstract

Introduction: Breast cancer is one of the most common malignancies. Early diagnosis and prevention usually lead to a good prognosis and a better survival rate. Despite significant advancements in the prevention, there is still a lack of effective therapies against particular subtypes. A complex interplay takes place between HIF-1α (hypoxia-inducible factor 1-alpha) and MYC, which reprograms the metabolism of cancer cells and promotes tumour cell growth, progression, and therapy resistance.

Aim: To study the immunohistochemical expression of HIF-1α and c-MYC in breast carcinoma cases and their correlation with clinicopathological parameters like age, tumour grade, tumour stage, nodal status and molecular subtypes, lymphovascular invasion, and perineural invasion, and their association with each other.

Materials and methods: A total of 61 cases of histopathologically confirmed invasive breast carcinoma were studied. Clinical and paraclinical characteristics were recorded. Immunohistochemistry for HIF-1α and c-MYC was done. HIF-1α expression was categorised as negative, low, and high, while c-MYC expression was scored as negative and positive. The data were analysed using univariate description and bivariate analysis, applying the chi-square test.

Result: The age ranged from 31 to 81 years, with a mean of 52.9 ± 12.9 years. HIF-1α expression was significantly correlated with the grade of the tumour (p = 0.035). However, no significant association was obtained between HIF-1α and age, lymphovascular invasion (LVI), perineural invasion (PNI), tumour stage, nodal status, proliferative index, and molecular subtypes. Our study did not find any significant correlation between c-MYC expression with histologic grade, LVI, PNI, tumour stage, nodal status, molecular subtypes, and HIF-1α expression.

Conclusion: An increase in HIF-1α expression correlated with aggressive behaviour of cancer and a higher likelihood of metastatic tumours. HIF-1α inhibitors can be considered as a newer therapeutic target.

## Introduction

In 2022, female breast cancer was the second leading cause of global cancer incidence and the fourth leading cause of cancer mortality worldwide [1]. Recently, there has been a higher proportion of the disease occurring at a younger age in Indian women, as compared to the West [2]. Early disease diagnosis can lead to a good prognosis and high survival rate [3]. Despite significant advancements in the prevention of breast cancer in the last few years, there is still a lack of effective therapies against particular subtypes. This has led to the development of its molecular classification. Eventually, this has improved the treatment modalities and subsequently the overall survival of breast cancer patients. Some subtypes, like the triple-negative breast carcinoma, still have a poor prognosis, and there are limited options for an optimal targeted therapy. The development of targeted drug design has grown quickly, hence providing numerous agents that target these markers for in vivo investigation in animal models as well as clinical studies [4].

HIF-1α (hypoxia-inducible factor 1-alpha) is a tightly regulated component of the transcriptional factor hypoxia-inducible factor (HIF). It regulates tumour cells by adapting and responding to stress factors like tumour hypoxia [5]. It has been found to be instrumental in tumour progression and aggressiveness [5]. It is often overexpressed and activated in many human cancers. HIF-1α plays vital roles in various aspects of breast carcinoma biology, such as stem cell maintenance, metabolic reprogramming, angiogenesis, invasion, epithelial-mesenchymal transition, metastasis, and resistance to radiation and chemotherapy [6]. The transcription factor c-MYC is a crucial regulator of the cell cycle by driving quiescent cells into the cell cycle [7]. Deregulated MYC promotes tumour growth and metastasis [8].

Rationale

A complex interplay occurs between MYC and HIF-1α, which reprograms the metabolism of cancer cells and promotes tumour cell growth, progression, and therapy resistance [9]. In animal models of breast carcinoma, HIF-1 inhibitors such as acriflavine and digoxin have demonstrated promising potential therapeutic effects by inhibiting the growth, invasion, vascularisation, and spread of tumours. These may be useful in treating triple-negative breast carcinomas due to the significant activation of the HIF-1 pathway in these tumours.

Knowledge gap

To the best of our knowledge, HIF-1α and c-MYC expression and their correlation with clinicopathological parameters and molecular subtypes of breast carcinoma have not been studied in the Indian subcontinent. Detection of HIF-1α might suggest a higher stage tumour, poor prognosis, and resistance to therapy. HIF-1α inhibitors may be proposed as a possible treatment strategy for such patients.

The aim and objective of the study was to study the immunohistochemical expression of HIF-1α and c-MYC in breast carcinoma and their correlation with clinicopathological parameters like age, tumour grade, tumour stage, nodal status and molecular subtypes, lymphovascular invasion, and perineural invasion, and their association with each other.

## Materials and methods

Study design and setting

This cross-sectional study was conducted in the pathology department of our institution for a period from March 2022 to February 2024, and was approved by the Institutional Ethics Committee (KIIT/KIMS/IEC/1024/2022). Histologically proven breast carcinoma cases were included in the study by a consecutive sampling method. Inadequately preserved specimens, core biopsy, patients who have received chemotherapy or radiotherapy before surgery, and poorly fixed tissue unsuitable for immunohistochemistry (IHC) were excluded from our study. Clinicopathological parameters like age, sex, tumour site, histopathological grade, tumour stage, lymphovascular invasion (LVI), perineural invasion (PNI), lymph node status, proliferative index, and molecular subtype were noted for each case.

Sample collection and histopathological processing

A total of 79 breast carcinoma cases were identified. After applying exclusion criteria, nine cases of preoperative chemotherapy, seven core biopsy cases, and two partially autolysed samples were excluded. A total of 61 cases of histologically proven breast carcinoma were included in the study. Grossing was done according to the College of American Pathologists (CAP) guidelines. Tissues were processed following standard protocols for paraffin embedding. Sections of 3-4 µm thickness were prepared from the microtome and stained with haematoxylin and eosin (H&E) for histopathological evaluation under light microscopy. Histopathological diagnosis with grading was done according to the CAP protocol.

IHC staining and interpretation

HIF-1α (Clone: rabbit polyclonal antibody, acquired from Quartett (Biocyc), Berlin, Germany), c-MYC (Clone: rabbit monoclonal antibody against c-Myc QR061, acquired from Quartett (Biocyc)), estrogen receptor (ER) (Clone: rabbit monoclonal antibody - Clone QR013, Quartett), progesterone receptor (PR) (Clone: rabbit monoclonal antibody - Clone QR014, Quartett, Germany), Her2Neu (Clone: rabbit monoclonal antibody - Clone QR003, Quartett), and Ki-67 (Clone: rabbit anti-human Ki-67 monoclonal antibody - Clone QR015, Quartett) ready to use clones were immunohistochemically evaluated by secondary labelling method on formalin-fixed, paraffin-embedded tissue slices that were 3-5 μm. Antigen retrieval from the tissue sections was performed by adding the proteolytic enzyme, followed by application of wet heat in a microwave oven for six cycles (total 18 minutes). An unconjugated primary antibody was added to the retrieved antigen, followed by a secondary antibody, which was conjugated with horse radish peroxidase (HRP) enzyme labelled to a polymer (dextran chain). The chromogen DAB (3,3'-diaminobenzidine) was oxidised in the presence of hydrogen peroxide, releasing nascent oxygen, which imparts a brown colour to the tissue.

Positive tissue controls for the histopathological section were used as controls. External positive control for HIF-1α and C-MYC was prepared from congested spleen and colon carcinoma, respectively. External positive control slides were prepared from paraffin-embedded tissues, and the same IHC method was repeated. Negative control was prepared by omitting the primary antibody step, and was instead treated with buffered saline. Two pathologists who remained blind to the clinical data independently assessed the immunohistochemical staining by interobserver agreement (kappa = -0.93). The discrepant cases were reviewed and re-evaluated over a multiheaded microscope with a consensus observation.

**Figure 1:**
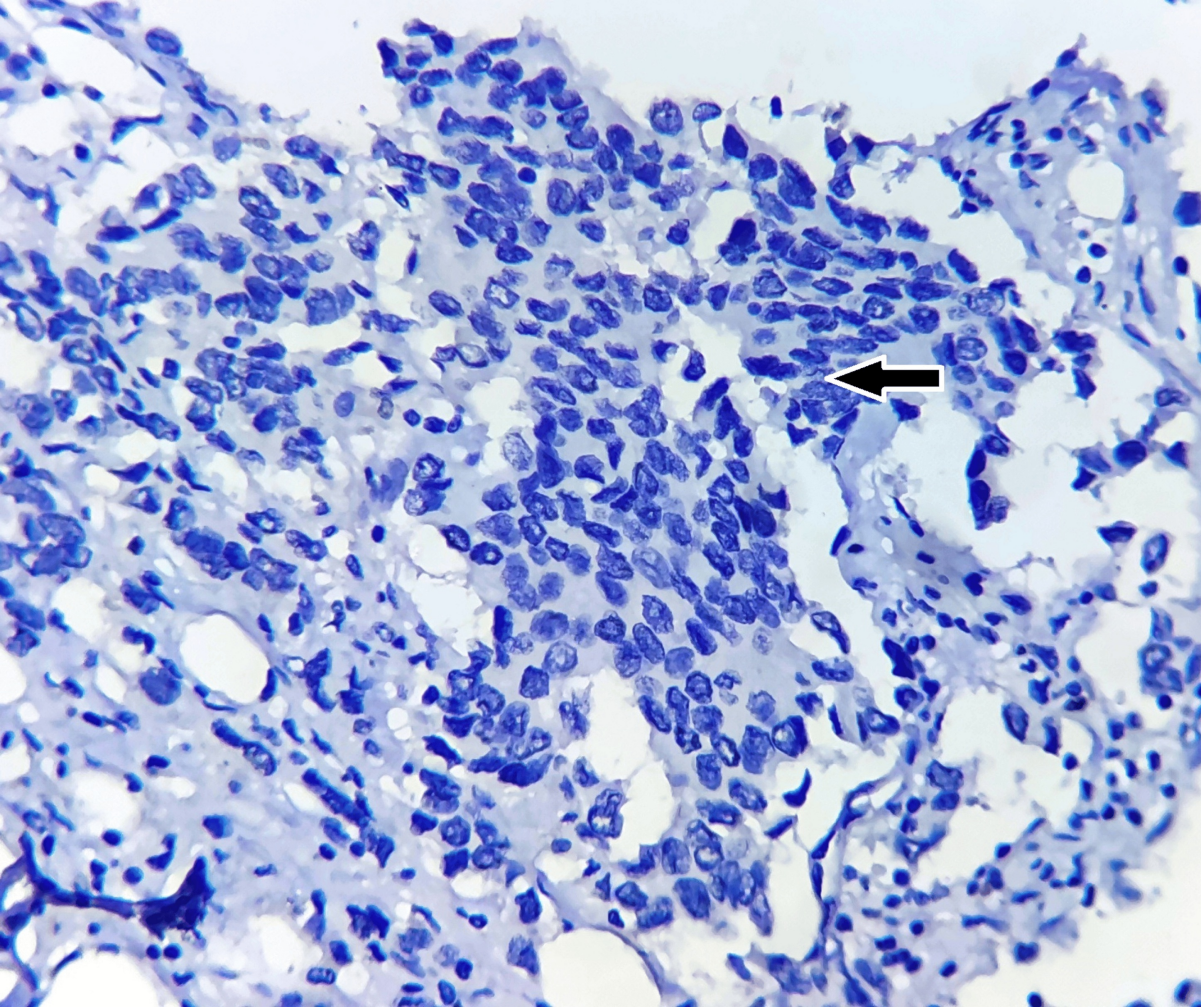
Negative nuclear expression of HIF-1α (black arrow), with immunohistochemistry magnification of 200x, DAB staining, counterstained by haematoxylin. HIF-1α: hypoxia-inducible factor 1-alpha; DAB: 3,3'-diaminobenzidine. The image is original and obtained at Kalinga Institute of Medical Sciences (KIMS), Bhubaneswar.

**Figure 2:**
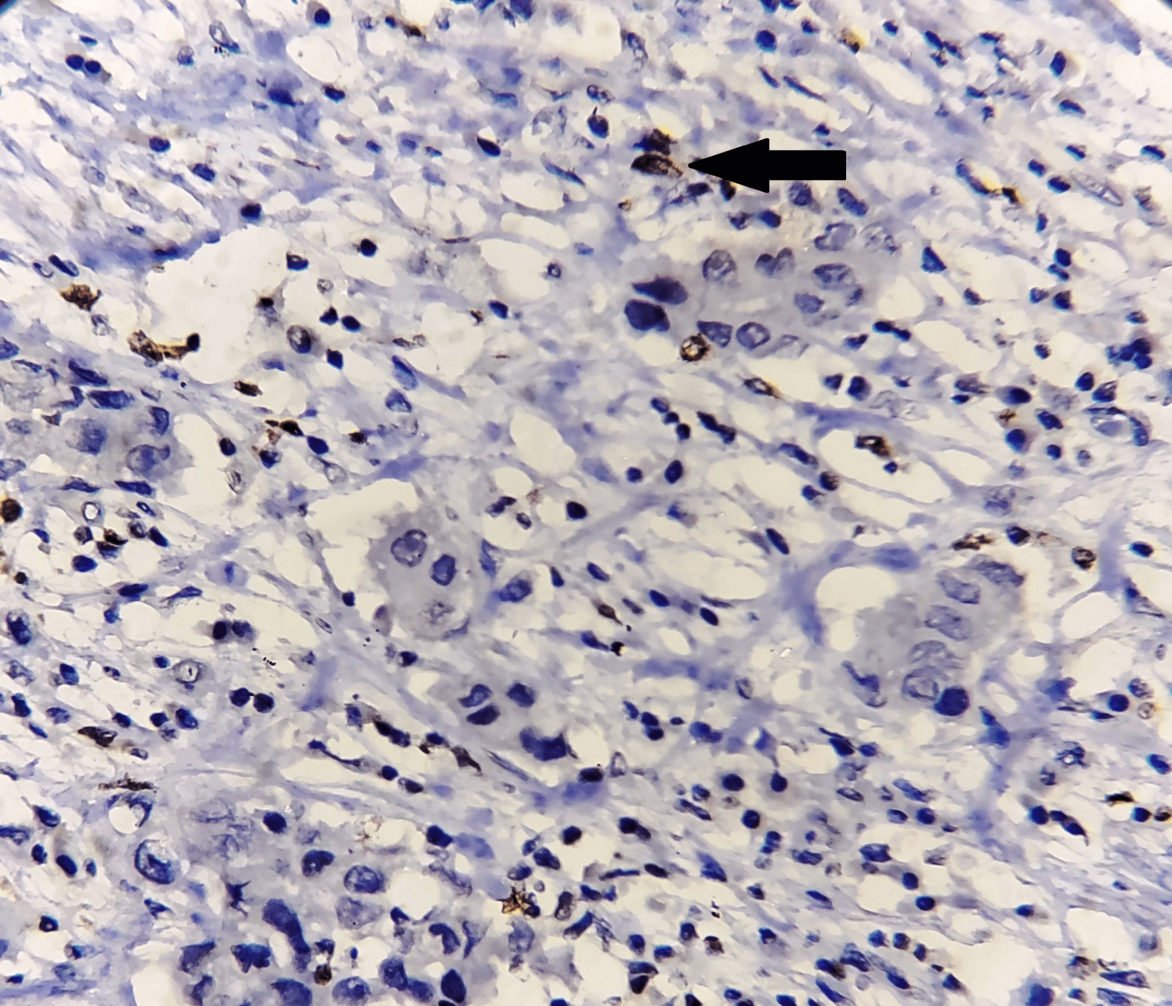
Low nuclear expression of HIF-1α (black arrow), with immunohistochemistry magnification of 200x, DAB staining, counterstained by haematoxylin. HIF-1α: hypoxia-inducible factor 1-alpha; DAB: 3,3'-diaminobenzidine. The image is original and obtained at Kalinga Institute of Medical Sciences (KIMS), Bhubaneswar.

**Figure 3:**
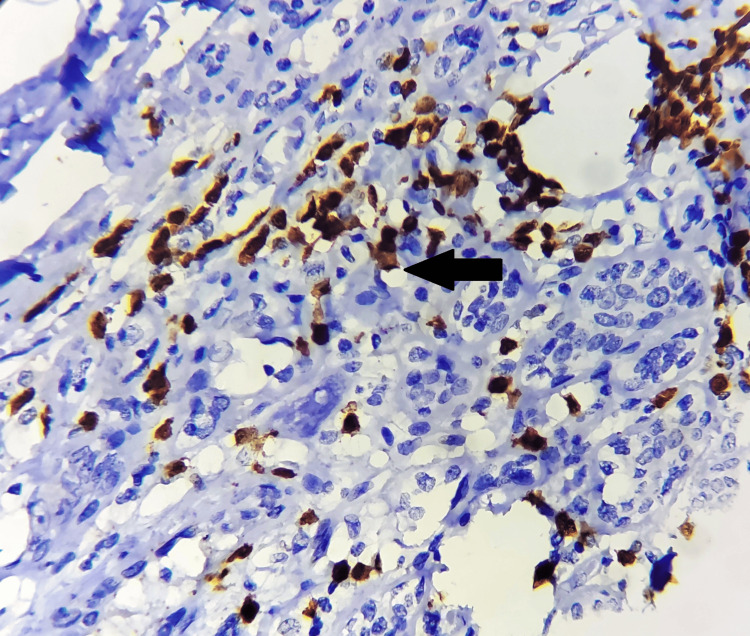
Strong nuclear expression of HIF-1α (black arrow), with immunohistochemistry magnification of 200x, DAB staining, counterstained by haematoxylin. HIF-1α: hypoxia-inducible factor 1-alpha; DAB: 3,3'-diaminobenzidine. The image is original and obtained at Kalinga Institute of Medical Sciences (KIMS), Bhubaneswar.

Ki-67 analysis was done in the hotspot area, counting around 1000 cells, and the percentage of positivity was taken. For HIF-1α analysis, nuclear staining intensity and percentage were studied. Tumour-cell immunopositivity was scored as both the number of HIF-1α-positive cells (nuclear staining) and the intensity: (-), not detected; (+), <1% positive cells; +, 1-10% weakly to moderately stained cells; ++, 1-10% intensively stained cells or 10-50% weakly stained cells; +++, 10-50% positive cells with moderate to marked staining; ++++, >50% positive cells. The six grades of staining were reformed to three grades: negative (0/(+)); low (+/++); and high (+++/++++), depicted in Figures 1-3 [5].

**Figure 4:**
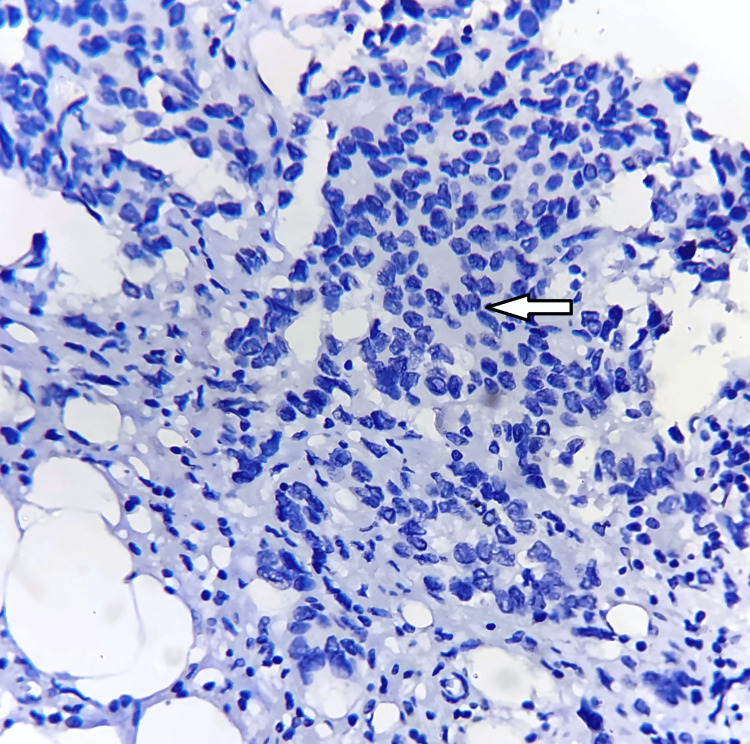
Negative nuclear expression of c-Myc (white arrow), with immunohistochemistry magnification of 200x, DAB staining, counterstained by haematoxylin. DAB: 3,3'-diaminobenzidine. The image is original and obtained at Kalinga Institute of Medical Sciences (KIMS), Bhubaneswar.

**Figure 5:**
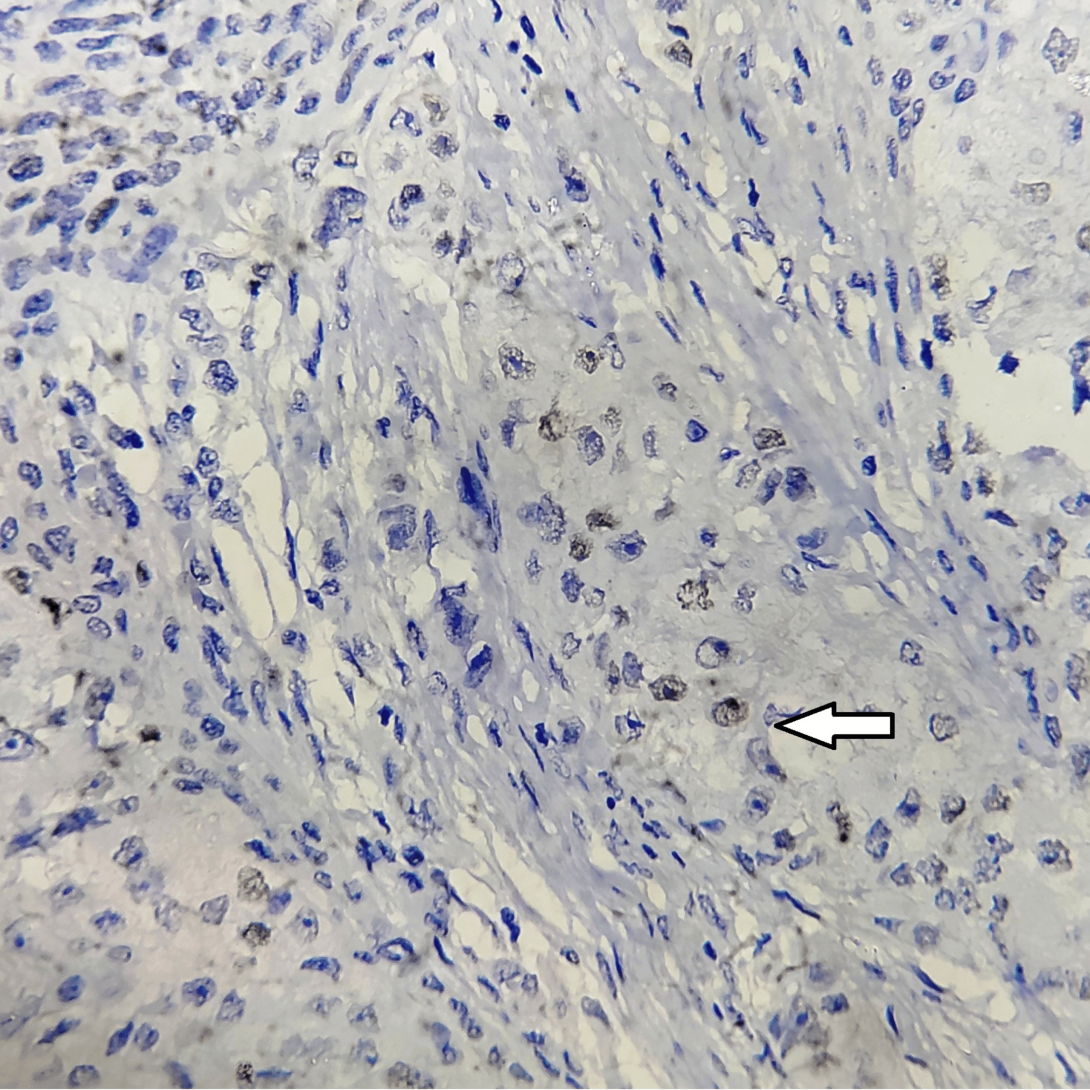
Positive nuclear expression of c-Myc (white arrow), with immunohistochemistry magnification of 200x, DAB staining, counterstained by haematoxylin. DAB: 3,3'-diaminobenzidine. The image is original and obtained at Kalinga Institute of Medical Sciences (KIMS), Bhubaneswar.

For c-MYC analysis, based on intensity and the number of cells staining, respectively, intensity scores were assigned 0, 1, 2, and 3, and percentage scores were assigned as 1 = 1 -25, 2 = 26-50, 3 = 51-75, and 4 = 76-100%. For IHC, an intensity score of >1 was assigned as high, and a percentage score of >3 was categorised as high, i.e., positive expression, and others as negative expression, depicted in Figures 4, 5 [10].

Statistical analysis

Statistical analysis was done using Microsoft Excel (Microsoft Corporation, Redmond, WA) and SPSS Statistics version 26 (IBM Corp., Armonk, NY) [11]. Categorical variables were summarised as frequencies and percentages, while continuous variables were expressed as mean ± standard deviation (SD). The chi-square test was applied to evaluate the association between HIF-1α and c-Myc with clinicopathological parameters. A p-value of less than 0.05 was considered significant.

## Results

This study analysed 61 cases of breast carcinoma. The distribution of cases according to clinicopathological parameters is depicted in Table 1. The age of the patients in this study ranged from 31 to 81 years, with a mean age of 52.9 ± 12.9 years, and the median age was 52 years. The most commonly observed cases were of grade II and III, with 26 cases (42.6%) each. The majority of tumours belonged to the T2 stage, with 37 cases (60.6%), and the highest proliferative index, with 57 cases (93.4%). Among the various molecular subtypes, luminal B HER2-negative comprised the maximum number of cases, i.e., 23 cases (37.8%).

**Table 1:** Distribution according to clinicopathological parameters. This table summarises the clinicopathological parameters of cases included in the study. Age was extracted from medical records at the time of diagnosis. Histological type, grade, LVI, and PNI were determined from biopsy specimens. Molecular subtyping was done by immunohistochemistry examination of the biopsy specimen. HIF-1α: hypoxia-inducible factor 1-alpha; LVI: lymphovascular invasion; PNI: perineural invasion.

Parameters		No. (%)
Age	30-45	18 (29.5)
	45-60	28 (45.9)
	60+	15 (24.6)
Histopathological grade	Grade I	9 (14.7)
	Grade II	26 (42.6)
	Grade III	26 (42.6)
LVI	Present	33 (54.1)
	Absent	28 (45.9)
PNI	Present	20 (32.8)
	Absent	41 (67.2)
T stage	T1 (≤2 cm)	10 (16.3)
	T2 (2-5 cm)	37 (60.6)
	T3 (>5 cm)	09 (14.7)
	T4	05 (8.1)
Lymph node status	Uninvolved	29 (47.5)
	Involved	32 (52.5)
Proliferative index (Ki-67)	<14	04 (6.6)
	≥14	57 (93.4)
Molecular subtypes	Luminal A	3 (4.9)
	Luminal B HER2 negative	23 (37.8)
	Luminal B HER2 positive	15 (24.6)
	HER2 enriched	7 (11.4)
	Triple negative	13 (21.3)

Among 61 breast carcinoma cases, 28 cases (45.9%) showed HIF-1α expression. Out of which, 13 cases (21.4%) revealed high expression and 15 cases (24.5%) demonstrated low expression. The maximum number of cases, i.e., 56 cases (91.9%), showed negative c-Myc expression, with only five cases (8.1%) being positive.

The most number of cases, i.e., 15 (45.4%), showed negative expression of HIF-1α in the age group of 46-60 years. But there was no correlation between HIF-1α and age group (p = 0.853). Among positive expressions of HIF-1α, low expression was noted in the maximum number, i.e., 12 cases (80.0%) of LVI-positive expression. There was no significant association found between LVI and HIF-1α (p = 0.068). The maximum number of high HIF-1α expression (11 cases, 84.6%) had no PNI identified. No significant association was found between PNI and HIF-1α (p = 0.295). The results are depicted in Table 2 (IBM SPSS version 26 was used).

**Table 2:** Association of HIF-1α with clinicopathological parameters. This table shows the association of HIF-1α expression in breast carcinoma with clinicopathological parameters. HIF-1α expression in breast carcinoma was studied by the immunohistochemistry method and then correlated with other variables. P-value <0.05 was considered significant. Chi-square test was used for calculating the p-value. HIF-1α: hypoxia-inducible factor 1-alpha; LVI: lymphovascular invasion; PNI: perineural invasion.

Parameters		Hif-1α expression			P-value
		Negative, n (%)	Low, n (%)	High, n (%)	
Age	30-45	11 (33.4%)	03 (20.0%)	04 (30.8%)	0.853
	46-60	15 (45.4%)	08 (53.3%)	05 (38.4%)	
	>60	7 (21.2%)	04 (26.7%)	04 (30.8)	
Histologic grade	Grade I	06 (18.2%)	02 (13.3%)	01 (7.7%)	0.035
	Grade II	19 (57.6%)	04 (26.7%)	03 (23.1%)	
	Grade III	08 (24.2%)	09 (60.0%)	09 (69.2%)	
LVI	Absent	18 (54.5%)	03 (20.0%)	07 (53.8%)	0.068
	Present	15 (45.5%)	12 (80.0%)	06 (46.2%)	
PNI	Absent	20 (60.1%)	10 (66.7%)	11 (84.6%)	0.295
	Present	13 (39.9%)	05 (33.3%)	02 (15.4%)	
Lymph node metastasis	N0	15 (45.5%)	06 (40.0%)	08 (61.5%)	0.762
	N1	11 (33.3%)	05 (33.3%)	04 (30.8%)	
	N2	02 (6.0%)	02 (13.3%)	01 (7.7%)	
	N3	05 (15.2%)	02 (13.3%)	0 (0.0%)	
Tumour stage	T1	05 (15.1%)	01 (6.7%)	04 (30.8%)	0.191
	T2	22 (66.7%)	11 (73.3%)	04 (30.8%)	
	T3	05 (15.1%)	01 (6.7%)	03 (23.1%)	
	T4	01 (3.1%)	02 (13.3%)	02 (15.3%)	
Ki-67	<14%	02 (6.1%)	01 (6.7%)	01 (7.7%)	0.980
	≥14%	31 (93.9%)	14 (93.3%)	12 (92.3%)	
Molecular subtype	Luminal A	02 (6.1%)	01 (6.7%)	00 (0.0%)	0.094
	Luminal B Her2 negative	13 (39.4%)	08 (53.3%)	02 (15.3%)	
	Luminal B Her2 positive	11 (33.3%)	01 (6.7%)	03 (23.1%)	
	Her2 enriched	01 (3.0%)	02 (13.3%)	04 (30.8%)	
	Triple negative	06 (18.2%)	03 (20.0%)	04 (30.8%)	

**Figure 6:**
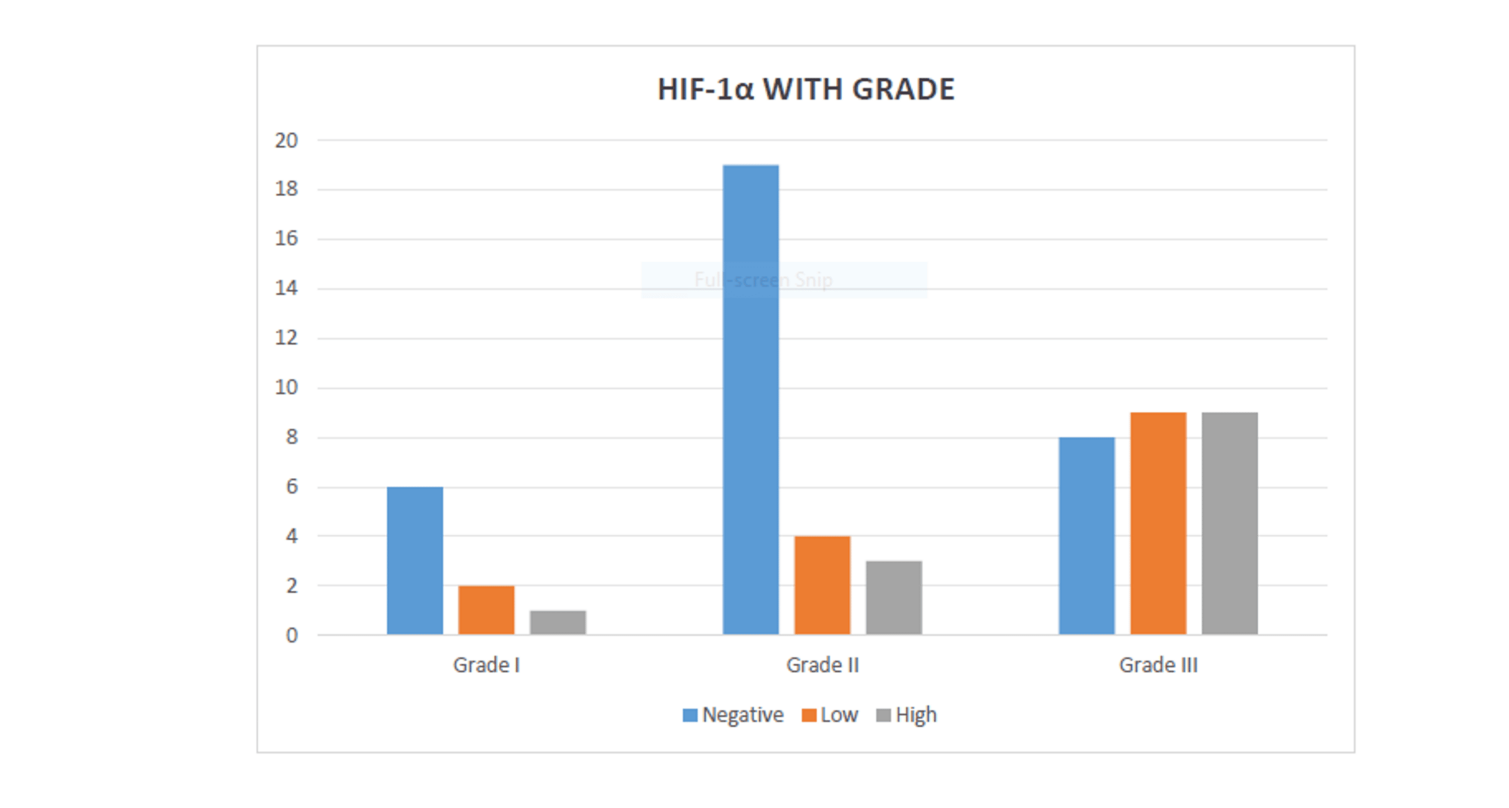
Association of histological grade with HIF-1α expression. A statistically significant correlation was found between histological grade and expression of HIF-1α (p = 0.035). HIF-1α: hypoxia-inducible factor 1-alpha.

The largest number of negative HIF-1α expression was noted in 19 cases (57.6%), which belonged to grade II. The maximum number of low (nine cases, 60.0%) and high (nine cases, 69.2%) HIF-1α expressions were seen in grade III. A statistically significant correlation was found between histological grade and expression of HIF-1α (p = 0.035) (Figure 6). There was no statistical significance seen between tumour stage, nodal status, and HIF-1α expression (p = 0.191 and 0.762, respectively). No significant correlation was found between HIF-1α expression and the molecular subtypes and proliferative index (p = 0.094 and 0.980).

The present study also explored the association between c-Myc expression and age, tumour grade, tumour stage, nodal status, and molecular subtypes, as depicted in Table 3 (IBM SPSS version 26 was used). There was no significant correlation found with age (p = 0.799), grade (p = 0.549), stage (p = 0.207), lymph node involvement (p = 0.815), and molecular subtype (p = 0.735).

**Table 3:** Association of c-Myc with clinicopathological parameters. This table shows the association of c-Myc expression in breast carcinoma with clinicopathological parameters. c-Myc expression in breast carcinoma was studied by the immunohistochemistry method and then correlated with other variables. P-value <0.05 was considered significant. Chi-square test was used for calculating the p-value. LVI: lymphovascular invasion; PNI: perineural invasion.

Parameters		c-Myc expression		P-value
		Low (n, %)	High (n, %)	
Age	30-45	17 (30.4%)	01 (20.0%)	0.799
	46-60	25 (44.6%)	03 (60.0%)	
	>60	14 (25.0%)	01 (20.0%)	
Histologic grade	Grade I	09 (16.0%)	00 (0.0%)	0.549
	Grade II	24 (42.9%)	02 (40.0%)	
	Grade III	23 (41.1%)	03 (60.0%)	
LVI	Absent	25 (44.6%)	03 (60.0%)	0.848
	Present	31 (55.4%)	02 (40.0%)	
PNI	Absent	38 (67.9%)	03 (60.0%)	1.000
	Present	18 (32.1%)	02 (40.0%)	
Lymph node status	N0	27 (48.2%)	02 (40.0%)	0.815
	N1	18 (32.1%)	02 (40.0%)	
	N2	05 (8.9%)	00 (0.0%)	
	N3	06 (10.8%)	01 (20.0%)	
Tumour stage	T1	10 (17.9%)	00 (0.0%)	0.207
	T2	35 (62.5%)	02 (40.0%)	
	T3	07 (12.5%)	02 (40.0%)	
	T4	04 (7.1%)	01 (20.0%)	
Molecular subtype	Luminal A	03 (5.3%)	00 (0.0%)	0.735
	Luminal B Her2 negative	22 (39.3%)	01 (20.0%)	
	Luminal B Her2 positive	14 (25.0%)	01 (20.0%)	
	Her2 enriched	06 (10.7%)	01 (20.0%)	
	Triple negative	11 (19.7%)	02 (40.0%)	

There was also no significant association found between HIF-1α and c-Myc expression in breast carcinoma cases (p = 0.567) (Table 4) (IBM SPSS version 26 was used). Statistical power is limited due to a small sample size.

**Table 4:** Association of HIF-1α with c-Myc. P-value <0.05 was considered significant. Chi-square test was used for calculating the p-value. HIF-1α: hypoxia-inducible factor 1-alpha.

		HIF-1 alpha			Total No. (%)	P-value
		Negative	Low	High		
		n (%)	n (%)	n (%)		
C-myc	Negative	31 (93.9%)	14 (93.3%)	11 (84.6%)	56 (91.9)	0.566
	Positive	02 (6.1%)	01 (6.7%)	02 (15.4%)	05 (8.1)	

## Discussion

Breast carcinoma poses a significant management challenge for clinicians as it is a heterogeneous malignancy with different molecular profiles and biological behaviour [12]. Various clinical, pathological, and genetic factors are considered for estimating the prognosis and determining the most appropriate therapeutic regimen [13]. However, there are many limitations to currently available treatment regimens; therefore, the need to expand treatment modalities still persists.

Among the emerging treatment options, molecular targeted therapy is a promising field. HIF-1α and its pathways are investigated as potential molecular targets for many cancers due to their involvement in various pathogenetic mechanisms. Hence, it is currently attracting attention for research regarding its use in the management of breast cancer. Though many investigations focused on the role of HIF-1α in breast carcinoma, most of the studies were performed on breast cancer cell lines and experimental animal models. Our study is one of the few that have analysed the expression of HIF-1α in tissues of patients with breast carcinoma. We also studied the transcription factor C-MYC, which has been seen to be overexpressed in many malignancies through different mechanisms [14].

Our study revealed that most of the cases with HIF-1α expression (46.4%) were in the age group of 46-60 years, i.e., mostly elderly persons. There was no statistically significant correlation (p = 0.853), which was in concordance with studies by Bos et al. (p = 0.22), Nie et al. (p = 0.59), Gruber et al. (p = 0.38), and Yamamoto et al. [5,15-17]. However, Cui et al. (p = 0.002) showed a significant association of age with HIF-1α expression [18]. The present study revealed C-MYC expression to be negative in most cases in all age groups. As a result, no significant correlation was found. Cui et al. also revealed similar findings [18].

**Figure 7:**
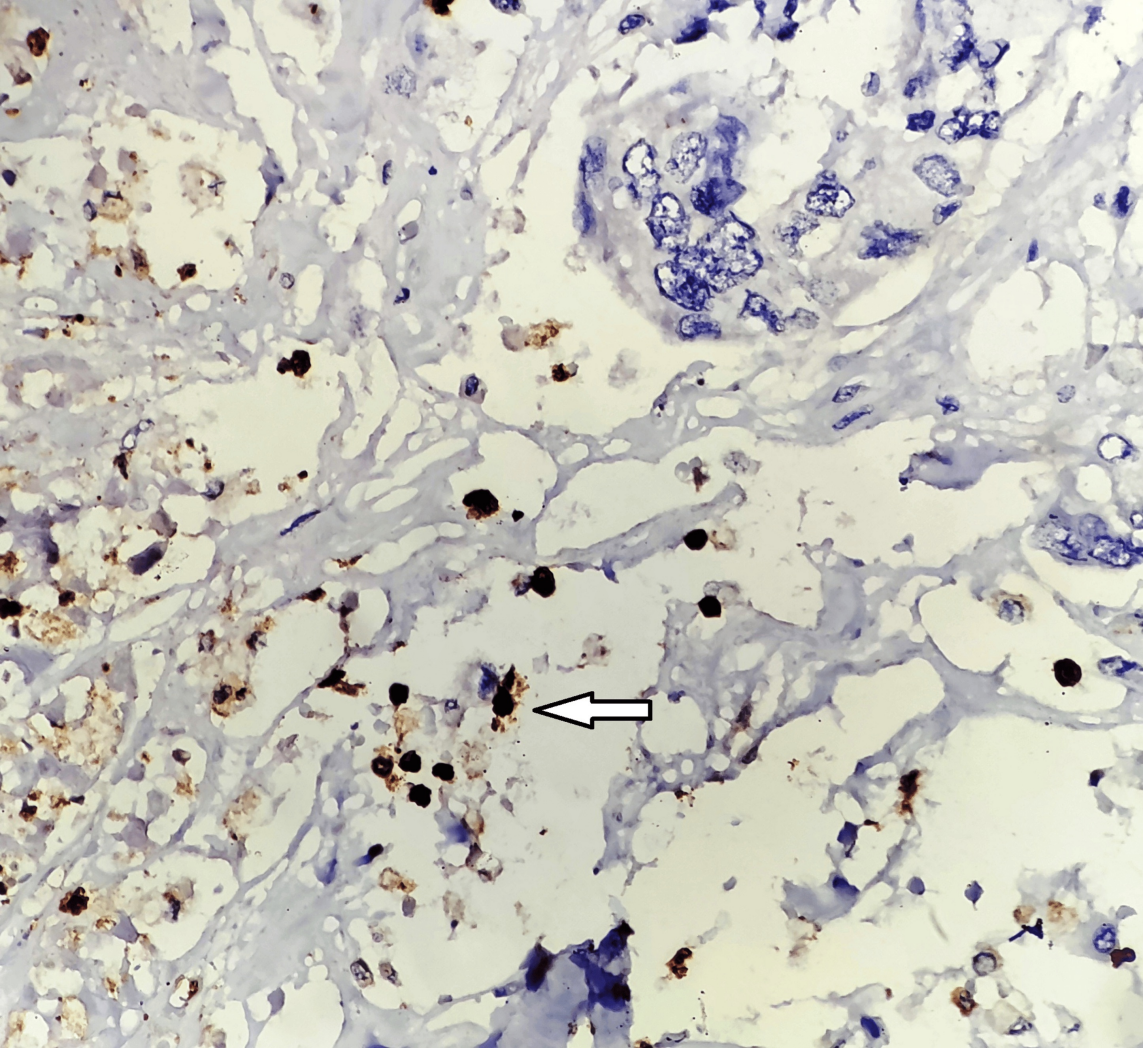
HIF-1α expression perinecrotic area (white arrow), immunohistochemistry 200x. HIF-1α: hypoxia-inducible factor 1-alpha. The image is original and obtained at Kalinga Institute of Medical Sciences (KIMS), Bhubaneswar.

**Figure 8:**
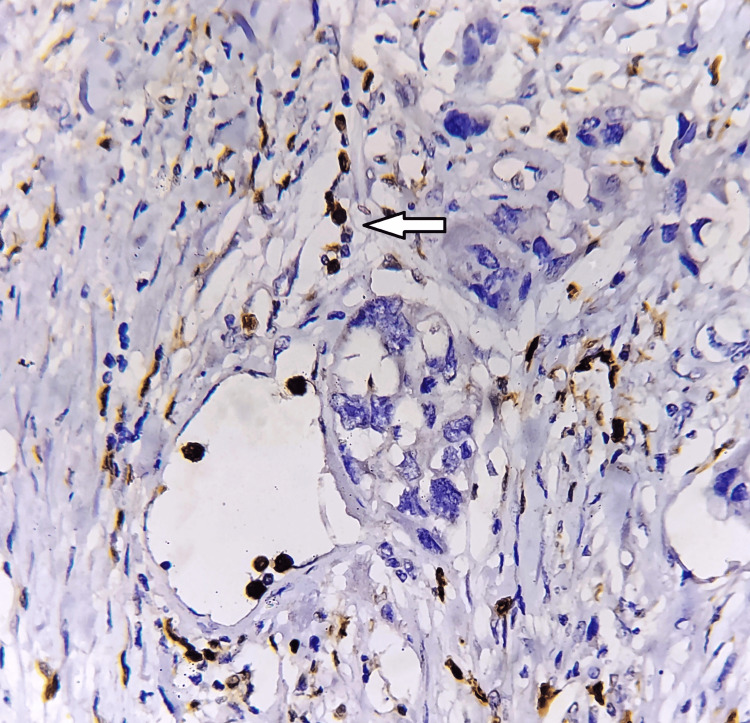
HIF-1α expression perivascular area (white arrow), immunohistochemistry 200x. HIF-1α: hypoxia-inducible factor 1-alpha. The image is original and obtained at Kalinga Institute of Medical Sciences (KIMS), Bhubaneswar.

Maximum positive HIF-1α expression cases were seen in grade III (64.28%), and it was found to be statistically significant (p = 0.035). Similar results were observed by Yamamoto et al. (p = 0.007), Bos et al. (p = 0.01), and Cui et al. (p = 0.016) [15,17,18]. However, Gruber et al. (p = 0.68) found no statistical significance [5]. It is a well-known fact that tumour grade increases with genetic alteration that leads to a change in the morphology of cells and nuclei, which is suitable for hypoxia. So, this suggests that HIF-1α expression increases with higher tumour grade. The present study showed the presence of LVI more in cases with positive HIF-1α expression. But there was no statistical significance (p = 0.068). However, a significant number of cases showed expression of HIF-1α in perinecrotic and perivascular areas (Figures 7, 8). Out of the HIF-1α-positive expression cases, most of them did not show PNI. No significant association was found with PNI. Our study has observed this, even though no study has been done earlier that investigated the correlation of PNI with HIF-1α expression.

In 80% of T4 tumours (4/5), positive expression of HIF-1α was noted. As the tumour stage increases, there was elevated HIF-1α expression with significant correlation, noted by others like Yamamoto et al. (p = 0.001) and Cui et al. (p = 0.005) [17,18]. Our study did not reveal similar findings (p = 0.191), which may be due to the very small number of T4 tumours. Similar results were also seen by Gruber et al. (p = 0.57) and Bos et al. (p = 0.35) [5,15]. Out of the 28 cases positive for HIF-1α expression in our study, 50% cases showed nodal metastasis, but it was not significantly correlated (p = 0.762). This was in concordance with studies done by Bos et al. (p = 0.39), Jogi et al. (p = 0.9), and Nie et al. (p = 0.22) [15,16,19]. However, Yamamoto et al. (p = 0.005) and Cui et al. (p = 0.007) in their study observed a significant correlation. HIF-1α expression was higher in node-negative cases compared to node-positive cases, which leads to prognostication [17].

In the current study, 45% of high proliferative index (≥14%) cases showed HIF-1α expression. No significant correlation was observed between the proliferative index and HIF-1α expression (p = 0.980). But studies by Bos et al. (p = <0.001), Yamamoto et al. (p = 0.0002), and Jogi et al. (p = 0.001) did obtain significant correlation [15,17,19]. In the present study, 14 cases with positive HIF-1α expression belonged to the luminal B subtype. Maximum cases of Her2-enriched tumours (six out of seven cases) showed HIF-1α positivity. There was no statistical significance in this. Similar findings were observed by Nie et al. (p = 0.867) [16]. Yamamoto et al., Gruber et al., and Bos et al. found statistical significance between PR and HIF-1α expression [5,15,17]. Yamamoto et al. also studied about correlation with ER. Bos et al. additionally correlated with HER2Neu. In our observation, HIF-1α-negative staining pattern was noted in ER-positive cases, i.e., luminal A & B subtype. HIF-1α positivity was seen in Her2-enriched and triple-negative breast cancers (TNBCs), which are aggressive phenotypes of breast cancer. This finding demonstrated elevated HIF-1α expression as a novel predictive factor.

Most cases were negative (56/61) for c-MYC. Looking at the histologic grade, c-MYC-positive expression was higher in high-grade tumours compared to that in low-grade tumours. Even though an increasing trend was noted among the c-MYC positive group, no statistically significant correlation was obtained between c-MYC expression and histological grade (p = 0.549). This may be due to a small sample size and c-MYC-positive tumours. Our findings are similar to the results of studies done by Dueck et al., Green et al., and Khan et al. [20-22]. Out of the low number of c-MYC-positive tumours, c-MYC expression was higher in LVI-negative cases. Among the LVI-positive cases, most were c-MYC negative. No statistical correlation was noted between LVI and c-MYC expression (p = 0.848). Currently, no studies are available that correlate HIF-1α and c-MYC expression with LVI in breast cancer.

Positive expression of c-MYC was noted in high-stage tumours. Our observation was not statistically significant, which may be due to a very small number of c-MYC-positive tumours. Dueck et al. observed an increasing trend and also a significant association with a p-value of 0.02 [20]. Positive c-MYC expression was not seen in luminal A. However, there was an increasing trend in aggressive molecular subtypes with the highest expression in TNBC in our study, but no statistical correlation was noted (p = 0.735). The study conducted by Khan et al. observed a statistically significant correlation with a p-value of <0.0001 [22].

Ours is one of the few studies to compare the immunohistochemical expression of HIF-1α and c-MYC in breast cancer. HIF-1α and c-MYC pathway is linked with regulation of metabolism and stimulation of angiogenesis, as a result leading to poor outcome [9,23,24]. With HIF-1α expression being significant in breast carcinoma, it is very important to understand the possible pathways through which it works, as they might be contributors to the failure of treatment. In our study, there was an increase in expression of c-MYC with an increase in HIF-1α expression. However, this was not found to be statistically significant (p = 0.566). Currently, no studies are available that correlate HIF-1α and c-MYC expression with each other in breast carcinomas.

A small sample size and a smaller number of c-MYC-positive cases were our biggest limitations. It is a prospective study from a single institute, and perhaps was responsible for not reaching statistical significance. To validate the significance of HIF-1α, more studies with a larger patient population are required. Insufficiently uniform cut-off values and conventional measurement techniques were also limitations.

Despite these demerits, measuring the expression of HIF-1α by immunohistochemistry can be a cheap and low-cost method appropriate for response assessment, prognostication, and prediction in locally advanced breast carcinomas. To establish HIF-1α evaluation as a credible biomarker in routine clinical practice, more prospective trials are required.

## Conclusions

To conclude, even though HIF-1α was expressed in less than 50% of the breast carcinoma cases included in our study, its expression increased in elderly persons, high histologic grade, size of tumour, and LVI-positive cases. So, HIF-1α can be a predictive marker. However, HIF-1α expression did not show any association with other parameters. The trend suggests an increase in HIF-1α expression correlated with aggressive behaviour of cancer and a higher likelihood of metastatic tumours. Hence, our findings highlight pathways that may merit further investigation of HIF-1α inhibitors as therapeutic targets. Studies done on a larger population may elucidate the association. Despite a significant correlation between HIF-1α and tumour grade in our study, it was not possible to conclusively establish the prognostic role of HIF-1α and c-MYC, given the limitations.

Exploration of newer pathways of cancer development, progression, and metastasis can help in developing newer targeted therapies. Just like the prognosis of HER2Neu-positive tumours has significantly improved by anti-Her2/neu agents, HIF-1α inhibitors could be potential treatment options in poor prognostic cases like triple-negative breast carcinomas, where no significant targeted therapy exists at present.
